# Telomerase activity in plasma cell dyscrasias

**DOI:** 10.1054/bjoc.2000.1655

**Published:** 2001-03

**Authors:** D Xu, C Zheng, S Bergenbrant, G Holm, M Björkholm, Q Yi, A Gruber

**Affiliations:** Department of Medicine, Division of Hematology, Karolinska Hospital, SE-171 76, Stockholm, Sweden

**Keywords:** hTERT, multiple myeloma, plasma cells, telomerase

## Abstract

Activation of telomerase is essential for in vitro cellular immortalization and tumorigenesis. In the present study, we investigated telomerase activation and its implications in plasma cell dyscrasias including monoclonal gammopathy of undetermined significance (MGUS), multiple myeloma (MM) and plasma cell leukaemia (PCL). All 5 patients with MGUS exhibited normal levels of telomerase activity in their plasma cells. Elevated telomerase activity was found in the samples from 21/27 patients with MM and 4/4 with PCL. In addition, 4 myeloma cell lines all expressed high levels of telomerase activity. The expression of telomerase reverse transcriptase (hTERT) and telomerase RNA template (hTER) was positively associated with the levels of telomerase activity in MM/PCL. Tankyrase expression was upregulated, concomitant with the induction of hTERT and activation of telomerase in MM/PCL. The present findings indicate that MGUS cells may not be immortalized and that activation of telomerase plays a role in the malignant transformation from MGUS to MM. © 2001 Cancer Research Campaign http://www.bjcancer.com


*British Journal of Cancer* (2001) **84**(5), 621–625
© 2001 Cancer Research Campaign
doi: 10.1054/ bjoc.2000.1655, available online at http://www.idealibrary.com on IDEAL

### Telomerase activity in plasma cell dyscrasias

**D Xu*, C Zheng*, S Bergenbrant, G Holm, M Björkholm, Q Yi# and A Gruber**

Department of Medicine, Division of Hematology, Karolinska Hospital, SE-171 76 Stockholm, Sweden

**Summary** Activation of telomerase is essential for in vitro cellular immortalization and tumorigenesis. In the present study, we investigated telomerase activation and its implications in plasma cell dyscrasias including monoclonal gammopathy of undetermined significance (MGUS), multiple myeloma (MM) and plasma cell leukaemia (PCL). All 5 patients with MGUS exhibited normal levels of telomerase activity in their plasma cells. Elevated telomerase activity was found in the samples from 21/27 patients with MM and 4/4 with PCL. In addition, 4 myeloma cell lines all expressed high levels of telomerase activity. The expression of telomerase reverse transcriptase (hTERT) and telomerase RNA template (hTER) was positively associated with the levels of telomerase activity in MM/PCL. Tankyrase expression was upregulated, concomitant with the induction of hTERT and activation of telomerase in MM/PCL. The present findings indicate that MGUS cells may not be immortalized and that activation of telomerase plays a role in the malignant transformation from MGUS to MM. © 2001 Cancer Research Campaign http://www.bjcancer.com

**Keywords**: hTERT; multiple myeloma; plasma cells; telomerase

It has been suggested that multiple myeloma (MM), a clonal B-cell malignancy involving plasma cells, arises from a typical stepwise neoplastic transformation (Hallek et al, 1998). With the accumulation of progressive genetic events that favour proliferative advantages and the expansion of the mutant cell clone, MM proceeds from non-proliferating normal plasma cells to those with extended lifespan, then with potential of immortal growth, and finally with a neoplastic phenotype. In relation to clinical progression, immortalization and transformation steps generally correspond to monoclonal gammopathy of undetermined significance (MGUS) and MM, respectively (Hallek et al, 1998).

The process of MM pathogenesis as indicated above is apparently similar to the in vitro immortalization and transformation model represented by viral and cellular oncogene-transformed somatic cells. It has been proposed that activation of telomerase and subsequent stabilization of telomere length is a critical step for in vitro cellular immortalization (Harley et al, 1994). Telomerase, an RNA-dependent polymerase, synthesizes telomere TTAGGG repeat sequences at the ends of chromosomes required for the integrity and stability of linear chromosomes (Harley et al, 1994). Normal somatic cells, generally lacking telomerase activity, display progressive attrition of telomeric DNA with each round of cell division since conventional DNA polymerase is unable to fully replicate linear DNA molecules and the cells eventually undergo senescence when a threshold telomere length is reached (Harley et al, 1994). Telomere shortening is thus suggested to act as a mitotic clock which records cellular replicative history and limits cellular proliferative lifespan. Accumulated evidence has demonstrated that most human tumour cells override the mitotic clock barrier and stabilize their telomere length via activating telomerase (Harley et al, 1994). The prevalence of telomerase activation has been observed not only in cultured immortal cell lines but also in the vast majority of human primary malignancies. The specific link of telomerase activation with cell immortalization and human cancers makes it a useful diagnostic marker and an attractive target for cancer therapy.

Besides telomerase, telomere length is apparently regulated by some other factors. A novel poly (ADP-ribose) polymerase named tankyrase has recently been identified and it may allow telomerase to approach the chromosome ends and to elongate telomeres (Smith et al, 1998). Although tankyrase is widely expressed in human normal tissues/cells, the highest level of tankyrase mRNA was detected in testis that also exhibits strong telomerase activity. It is currently unclear how tankyrase is regulated and whether it plays a role in human primary malignancies.

The spectrum MGUS-MM-PCL provides an example to analyse telomerase activation during in vivo tumorigenesis. Given that activation of telomerase is a prerequisite for cellular immortalization, it would be expected that both MGUS and MM exhibit telomerase activity based on the pathogenesis model of MM suggested by Hallek and co-workers (1998). To test this hypothesis, we examined telomerase activity and its implications in plasma cells from patients with MGUS, MM and PCL. In addition, the expression of the telomerase components telomerase reverse transcriptase (hTERT), telomerase RNA template (hTER) and tankyrase was assessed in the samples in order to elucidate their role in telomerase activation or regulation in plasma cell dyscrasias.

*Received 26 June 2000*
*Revised 28 September 2000*
*Accepted 30 November 2000*

*Correspondence to:* D Xu

*The first two authors contribute equally to this work.

#Current address: Myeloma and Transplantation Research Center, University of Arkansas for Medical Sciences, Little Rock, Arkansas, USA.

### MATERIALS AND METHODS

### Patients

The study was performed on bone marrow (BM) plasma cells from 5 patients with MGUS, 27 with MM (4, 8, and 15 with stages I, II and III, respectively) and on peripheral blood plasma cells from 4 patients with PCL. The median age of the patients was 67 years (range 40–80). In addition, BM aspirates of 5 adult patients with non-malignant disorders were included as controls. The clinical staging of MM was defined according to the criteria proposed by Salmon and Durie (Durie and Salmon, 1975). Two patients had a Bence Jones, and one a non-secretory MM. 13 MM, and one PCL patient had received chemotherapy before sampling.

### Bone marrow samples

BM was drawn into heparinized glass tubes, and mononuclear cells were isolated with Ficoll-Paque (Pharmacia, Uppsala, Sweden) gradient centrifugation and washed in phosphate buffered saline (PBS). The cells were either subjected to $CD38^{bright}$ subpopulation separation or frozen to –160˚C in a programmed freezer in 90% human AB serum and 10% dimethylsulphoxide (DMSO) and thereafter stored in liquid nitrogen. Prior to analysis, the frozen cells were rapidly thawn at 37˚C, resuspended in 100% FCS, centrifuged to remove DMSO and dead cells, and finally washed twice with ice-cold PBS. The viability of cells was above 90% as determined by trypan blue exclusion.

### $CD38^{bright}$ plasma cell isolation

CD38 was chosen as a marker for plasma or MM cells (Yi et al, 1997). $2–5 \times 10^7$ BM mononuclear cells, fresh or newly thawn, were incubated with CD38 monoclonal antibody conjugated with R-phycoerythrin (R-PE) and then sorted by Mini-Magnetic Cell Sorting kit (Miltenyi Biotec, Sunnyale, CA, USA) according to the manufacturer's recommendations. Earlier results showed that the purity of $CD38^{bright}$ cells was around 70% for samples with less than 15% plasma cells in BM and 80% or more for samples with ≥15% of plasma cells as determined by flow cytometry (Yi et al, 1997). In the present study, the purity of the sorted cells were examined in samples from 3 patients (2 MGUS and 1 MM) with 3.3%, 5% and 9% plasma cells in their BM, respectively. The $CD38^{bright}$ cell purity after sorting was 71%, 73% and 91%, respectively. The purified $CD38^{bright}$ and CD38 negative cells were stored at –80˚C until use.

### Cell lines and cell culture

The REH cell line, derived from a human pre-B lymphocytic leukaemia, and 4 myeloma cell lines Karpas-707, HL-4076, U266 and LP-1 (kindly provided by Professor K Nilsson, Uppsala University, Sweden) were grown in RPMI 1640 medium (Gibco, Glasgow, UK) supplemented with 10% fetal calf serum, 2 mM L-glutamine and 100 U $ml^{-1}$ penicillin at 37˚C in humidified 5% $CO_2$/95% air atmosphere.

### Telomerase activity assay

Cells ($2 \times 10^5$–$2 \times 10^6$) were suspended in ice-cold standard lysis buffer (Kim et al, 1994). Protein extraction and measurement was performed as described (Xu et al, 1996, 1998). A commercial telomerase PCR ELISA kit (Roche Diagnostics Scandinavia AB, Stockholm, Sweden), based on Telomeric Repeats Amplification Protocol (TRAP) introduced by Kim et al (1994), was used to determine telomerase activity in all samples in duplicate according to the manufacturer's protocol. In a prior study we demonstrated that input of 0.5 μg of protein with 28 cycle PCR amplification and 3 μl of the PCR products for ELISA detection gave optimal and reproducible signals within exponential phase for telomerase activity detection (Xu et al, 1998). The level of telomerase activity in samples or cell lines was expressed as the percentage of that in the same amount of protein derived from REH cells.

### RNA extraction, reverse transcription and RT-PCR

Total cellular RNA from isolated plasma cells was extracted using the Ultraspec™-II RNA kit (Biotecx Lab., Houston, TX, USA). RNA yield and purity were determined spectrophotometrically at 260/280 nm. cDNA was synthesized using random primers (N6) (Pharmacia, Uppsala, Sweden) and MMLV reverse transcriptase as described (Xu et al, 1998). PCR primers for tankyrase were: (Forward) 5′CCCGACTTCTTCCTCATCTTC3′ and (Reverse) 5′GCGTCCACCAGCCTCTTTA3′. The resulting PCR product was 210 bp fragments. cDNA derived from 50 ng RNA in total 25 μl reaction mixture ($1 \times$ PCR buffer, 0.25 mM $MgCl_2$, 0.25 mM each dNTP, 0.625 U Taq polymerase and 0.06 μM each primer) was amplified in DNA thermal cycler 9600 (Perkin Elmer) using 29 cycles (94˚C 30″, 60˚C 30″ and 72˚C 45″). PCR conditions and primers for hTERT and hTER were described elsewhere (Xu et al, 1998), and amplification cycles were 34 and 30 for hTERT and hTER, respectively. In addition, $\beta_2$-microglobulin ($\beta_2$-M) expression was used as a control for RNA loading and RT efficiency and amplified during 25 PCR cycles (94˚C for 30″, 60˚C for 30″ and 72˚C for 60″) (Xu et al, 1998). In each PCR, cDNA from REH cells and $H_2O$ were included as positive and negative controls, respectively. PCR products were resolved in 2% agarose gels stained with ethidium bromide, visualized in UV light and photographed. Volumetric integration of signal intensities was performed by using the NIH Image software (Version 1.61). The relative levels of each gene expression were arbitrary values calculated from the ratio of the target to β2-M.

### Statistical analyses

Unpaired Student *t*-test was used to compare differences of telomerase activity between two groups. A correlation of telomerase activity with the percentage of plasma cells in BM or with hTERT, hTER and Tankyrase expression was tested with a simple regression analysis. All statistical analyses were performed using StatView 4.5 software produced by Abacus Concepts Inc. (Berkeley, CA, USA). The level of significance was set to a $P$ value < 0.05.

### RESULTS

### Telomerase activity in BM mononuclear cells from control BM samples

Normal human peripheral blood mononuclear cells and BM mononuclear cells exhibit weakly detectable telomerase activity

© 2001 Cancer Research Campaign

(Norrback et al, 1996; Xu et al, 1996, 1998; Bechter et al, 1998). We have previously shown that telomerase activity in normal PBMCs ranged from 3–8% of that in REH cells, as determined using Telomerase PCR ELISA kit (Xu et al, 1998). Similar baseline levels of telomerase activity (0–9%) were found in BM mononuclear cells from 5 adult patients with non-malignant disorders (Figure 1). Accordingly, a telomerase activity value beyond 10% was defined as higher than normal.

### Telomerase activity in MM cell lines and in plasma cells from patients with MGUS, MM and PCL

All 4 MM cell lines Karpas-707, HL-4076, U266 and LP-1 cells expressed strong telomerase activity ranging 71–117% of that in REH cells. Telomerase activity was within normal levels in plasma cells from all 5 patients with MGUS. The 4 samples from patients with PCL varied between 54 and 78%. Telomerase activity varied considerably in the samples from patients with MM, 1–101%. 21 of the 27 samples from patients with MM had elevated telomerase activity (2/4, 5/8, and 14/15 of the samples from patients with stages I, II and III disease, respectively). It seemed that the patients with the advanced disease were more likely to have increased telomerase activity in their plasma cells (Stage III *vs* I and II, $P = 0.06$, Fisher's exact test). However, there was no difference in mean telomerase activity in stages I, II and III (35.8 ± 38.7, 23.8 ± 15.8 and 48.4 ± 32.5, respectively, mean ± SD). As seen in Figure 1, telomerase activity exhibited a pattern as following: PCL > MM > MGUS.

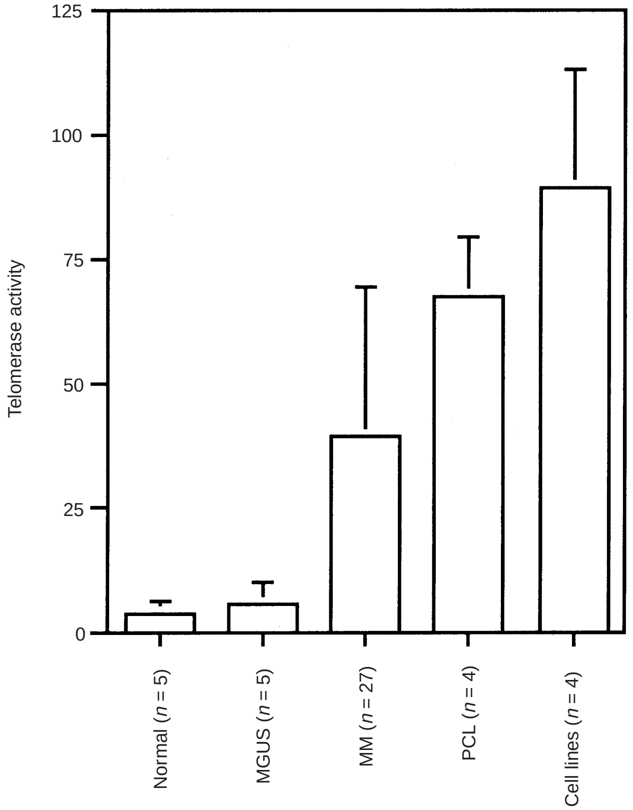

**Figure 1** Telomerase activity in normal bone marrow cells, monoclonal gammopathy of undetermined significance (MGUS), multiple myeloma (MM) and plasma cell leukaemia (PCL) and MM cell lines

The mean percentage of plasma cells in BM of patients with MM was 45.3 ± 30.9. There was no correlation between the percentage of plasma cells in bone marrow and telomerase activity ($r^2 = 0.039$, $P = 0.32$). In addition, no relationship was found between the size in the M-component and the levels of telomerase activity. Mean telomerase activity did not differ between samples from patients with MM/PCL, who received prior cytostatic treatment ($n = 14$) and those who did not ($n = 17$). 17 patients (4 stage II, 12 stage III and 1 PCL) received cytostatic treatment after sampling. Of 14 evaluable patients, 7 responded to chemotherapy with at least 25% reduction of the M-component. There was no significant difference in telomerase activity in plasma cells between patients with responding and unresponsive disease (32.6 ± 22.8 *vs* 53.7 ± 36.5, $P = 0.22$).

### Correlation of hTERT, hTER and tankyrase expression with telomerase activity in MM/PCL

To explore possible pathways that affect telomerase activity in plasma cells from patients with MM, the expression of telomerase components hTERT, hTER and newly identified tankyrase, implicated in telomerase function regulation, was determined in 10 samples from MM/PCL patients. The expression of hTERT mRNA was positively correlated with the levels of telomerase activity ($r^2 = 0.67$, $P = 0.004$, Figure 2A). There was also a significant positive association between hTER expression and telomerase activity ($r^2 = 0.45$, $P = 0.04$, Figure 2B). Interestingly, the expression of tankyrase appeared to have a weak correlation with telomerase activity ($r^2 = 0.29$, $P = 0.1$, Figure 2C), but was significantly related to the hTERT mRNA expression ($r^2 = 0.62$, $P = 0.007$).

### DISCUSSION

In the present study, we found elevated telomerase activity in 78% of MM, all of 4 PCLs and 4 MM cell lines. By contrast, telomerase activity was at normal levels in 5 MGUS samples. In a recent study of 31 patients with MM, telomerase activity was found to be considerably variable. High levels correlated to the presence of chromosomal changes (Orme et al, 1999). Cheryk et al (1999) showed that telomerase activity was present in plasma cells from 12 of 15 MM patients but absent in all 5 patients with MGUS. Taken together, the data suggest that the activation of telomerase may play a role in the malignant transformation from MGUS to MM.

The finding that telomerase activity was not increased in MGUS cells indicates that those cells are not immortal, which is apparently incompatible with the concept that MGUS cells are immortal as recently suggested by Hallek et al (1998) based on the genetic analysis data. Earlier studies indeed revealed that MGUS cells had already acquired several genetic aberrations (Hallek et al, 1998). These genetic events may affect the regulatory pathways that directly control cell birth/death, and consequently lead to extension of the lifespan of MGUS cells. However, the genetic mutations alone in MGUS are not indicators for acquisition of immortality, since no mutational events in genes responsible for cell growth control have, to date, been found to be capable of directly immortalizing normal human cells and immortalization may occur independently or require multiple genetic alterations (Harley et al, 1994). Characteristically, those early genetic events drive the cells to divide far beyond their normal replicative potential,

© 2001 Cancer Research Campaign 

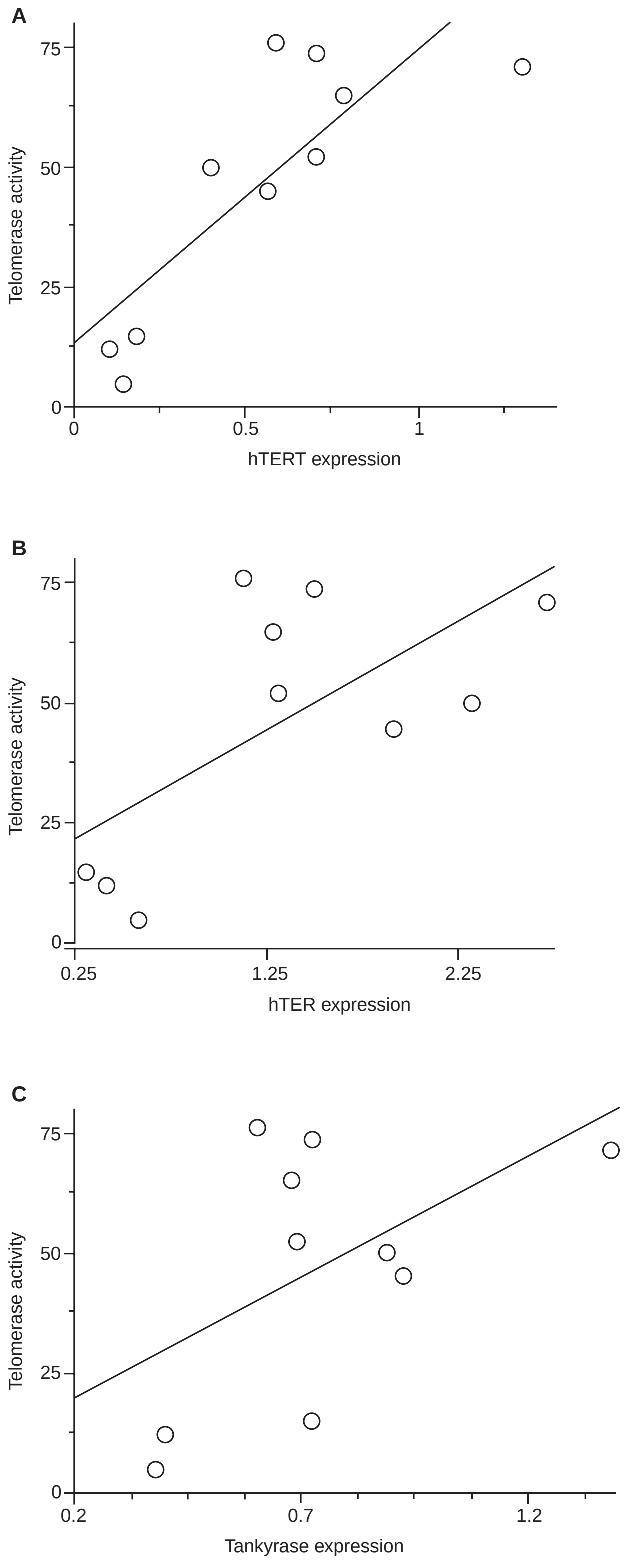

**Figure 2** Correlation of telomerase activity with the expression of telomerase reverse transcriptase (hTERT) ($r^2 = 0.67$, $P = 0.004$, (**A**), telomerase RNA template (hTER) ($r^2 = 0.45$, $P = 0.04$, (**B**), and tankyrase ($r^2 = 0.29$, $P = 0.1$, (**C**) in multiple myeloma (MM, $n = 8$) and in plasma cell leukaemia (PCL, $n = 2$)

thus exhaust their telomeric DNA sequences, and eventually trigger further genetic instability and cellular crisis. During this process, those cells whose telomerase is activated acquire immortal phenotype (Harley et al, 1994). MGUS cells have not extensively expanded yet, there is, thus, no selective advantages for them to mutate to the immortal phenotype at a pre-neoplastic stage. In addition, the clinical observation that only a small part of MGUS eventually progress into MM indicates that in most MGUS patients the mutant plasma cell clones are unable to circumvent the limitation imposed by telomere attrition on their replicative lifespan.

One may argue that as telomerase activation is not the only mechanism for maintenance of telomere length, it can not be ruled out that MGUS cells use alternative pathways (alternative lengthening of telomeres, ALT) to compensate for the shrinking of telomeric DNA sequences caused by cell replication (Bryan et al, 1997). ALT indeed operates in a small subset of tumour cell lines and in primary malignancies with non-epithelial in origin but is rare in haematological malignancies (Bryan et al, 1997). This hypothesis is apparently incompatible with the wide activation of telomerase observed in MM and PCL.

It is well documented that extremely short telomeres trigger cell crisis and subsequent activation of telomerase in in vitro immortalization and transformation model (Harley et al, 1994), however, defining the exact activation mechanism(s) remains a challenge. Numerous studies have indicated that telomerase activity is likely subject to multiple levels of control and regulation by different factors in different cellular contexts and circumstances. The core enzyme complex of telomerase consists of hTER and hTERT (Beattie et al, 1998), so the changes in hTER and hTERT expression will certainly lead to up- or down-regulation of telomerase activity. Previous investigations demonstrated that hTER expression, although readily detected in almost all human tissues/cells and not always correlated with telomerase activity, was upregulated with tumour progression (Avilion et al, 1996; Sallinen et al, 1997); whereas hTERT, the key component of telomerase, was a rate-limiting determinant of enzymatic activity in both normal and neoplastic cells (Meyerson et al, 1997). Consistent with these findings, we found that hTERT and hTER expression positively correlated to the levels of telomerase activity in MM and PCL. It is poorly understood how hTERT is derepressed during tumorigenesis at the moment. A detailed functional analysis on the hTERT promoter (Cong et al, 1999) will provide new insights into hTERT regulation and telomerase activation mechanisms in human malignancies including MM.

In addition, the regulation of telomerase may further operate at a functional level. Tankyrase allows telomerase to exert its elongation effect on telomeres. In the present study, tankyrase expression was found to be positively correlated with hTERT mRNA levels in MM/PCL cells. Similarly, we found a strong upregulation of tankyrase expression in activated T cells, concomitant with the induction of hTERT and telomerase activity (unpublished data). The data suggest a possible contribution of tankyrase upregulation in tumour development/progression.

In summary, we have demonstrated that activation of telomerase occurs widely in MM and PCL but not in MGUS. The observations suggest that telomerase activity, although not required for the pre-neoplastic lesion MGUS, plays a role during stepwise transformation and progression of MM. Upregulation of tankyrase expression, concomitant with telomerase activation observed in MM/PCL, may also contribute to tumorigenesis.

*© 2001 Cancer Research Campaign*

### ACKNOWLEDGEMENTS

This work was supported by the Swedish Cancer Society, the Cancer Society in Stockholm, Karolinska Institute Funds and Stockholm County Council.

### REFERENCES

Avilion AA, Piatyszek MA, Gupta J, Shay JW, Bacchetti S and Greider CW (1996) Human telomerase RNA and telomerase activity in immortal cell lines and tumor tissues. *Cancer Res* **56**: 645

Beattie TL, Zhou W, Robinson MO and Harrington L (1998) Reconstitution of telomerase activity *in vitro*. *Curr Biol* **8**: 177

Bechter OE, Eisterer W, Pall G, Hilbe W, Kuhr T and Thaler J (1998) Telomere length and telomerase activity predict survival in patients with B cell chronic lymphocytic leukemia. *Cancer Res* **58**: 4918

Bryan TM, Englezou A, Dalla-Pozza L, Dunham MA and Reddel RR (1997) Evidence for an alternative mechanism for maintaining telomere length in human tumors and tumor-derived cell lines. *Nat Med* **3**: 1271

Cheryk LA, Katzmann JA, Ahmann GJ, O'Kane DJ and Greipp PR (1999) Telomerase expression in plasma cells from patients with multiple myeloma but not monoclonal gammopathy of undetermined significance. *Proc Am Assoc Cancer Res* **40**: 264

Cong Y-S, Wen J and Bacchetti S (1999) The human telomerase catalytic subunit hTERT: organization of the gene and characterization of the promoter. *Hum Mol Genet* **8**: 137

Counter CM, Gupta J, Harley CB, Leber B and Bacchetti S (1995) Telomerase activity in normal leukocytes and in hematologic malignancies. *Blood* **85**: 2315

Durie BGM and Salmon SE (1975) A clinical staging system for multiple myeloma. Correlation of measured myeloma cell mass with presenting clinical features, response to treatment and survival. *Cancer* **36**: 842

Hallek M, Bergsagel PL and Anderson KC (1998) Multiple myeloma: increasing evidence for a multistep transformation process. *Blood* **91**: 3

Harley CB, Kim NW, Prowse KR, Weinreich SL, Hirsch KS, West MD, Bacchetti S, Hirte HW, Counter CM, Greider CW, Piatyszek MA, Wright WE and Shay JW (1994) Telomerase, cell immortality, and cancer. *Cold Spring Harbor Symposia Quantitative Biology* **59**: 307

Kim NW, Piatyszek MA, Prowse KR, Harley CB, West MD, Ho PLC, Coviello GM, Wright WE, Weinrich SL and Shay JW (1994) Specific association of human telomerase activity with immortal cells and cancer. *Science* **266**: 2011

Meyerson M, Counter CM, Eaton EN, Ellisen LW, Steiner P, Caddle SD, Ziaugra L, Beijersbergen RL, Davidoff MJ, Liu Q, Bacchetti S, Haber DA and Weinberg RA (1997) hEST2, the putative human telomerase catalytic subunit gene, is up-regulated in tumor cells and during immortalization. *Cell* **90**: 785

Norrback K-F, Dahlenborg K, Carlsson R and Roos G (1996) Telomerase activation in normal B lymphocytes and non-Hodgkin's lymphomas. *Blood* **88**: 222

Orme L, Shaughnessy J, Ayers G, Bailey C, Barlogie C, Barlogie B and Moore MAS (1999) Telomerase in myeloma is variable and correlates with certain disease characteristics. *Blood* **94** (Suppl.): 535a

Sallinen P, Miettinen H, Sallinen S-L, Haapasalo H, Helin H and Kononen J (1997) Increased expression of telomerase RNA component is associated with increased cell proliferation in human astrocytomas. *Am J Pathol* **150**: 1159

Smith S, Giriat I, Schmitt A and de Lange T (1998) Tankyrase, a poly (ADP-ribose) polymerase at human telomeres. *Science* **282**: 484

Xu D, Gruber A, Peterson C and Pisa P (1996) Suppression of telomerase activity in HL60 cells after treatment with differentiating agents. *Leukemia* **10**: 1354

Xu D, Gruber A, Peterson C and Pisa P (1998) Telomerase activity and the expression of telomerase components in acute myelogenous leukaemia. *Br J Haematol* **102**: 1367

Yi Q, Dabadghao S, Österborg A, Bergenbrant S and Holm G (1997) Myeloma bone marrow plasma cells: evidence for their capacity as antigen-presenting cells. *Blood* **90**: 1960

© 2001 Cancer Research Campaign 
